# Immunomodulatory effect of water soluble extract separated from mycelium of *Phellinus linteus* on experimental atopic dermatitis

**DOI:** 10.1186/1472-6882-12-159

**Published:** 2012-09-18

**Authors:** Ji Sun Hwang, Ho-Keun Kwon, Jung-Eun Kim, Jeonghae Rho, Sin-Hyeog Im

**Affiliations:** 1 School of Life Sciences and Immune Synapse Research Center, Gwangju Institute of Science and Technology (GIST), 261 Cheomdan-gwagiro, Buk-gu, Gwangju, 500-712, Korea; 2 Department of Microbiology and Immunobiology, Harvard Medical School, Boston, MA, 02115, USA; 3 Korea Food Research Institute, 516 Baekhyeon-Dong, Seongnam, 463-746, Republic of Korea

## Abstract

**Background:**

Complementary and alternative medicine (CAM) is becoming a popular treatment for modulating diverse immune disorders. *Phellinus linteus* (*P. linteus*) as one of the CAMs has been used to modulate cancers, inflammation and allergic activities**.** However, little evidence has been shown about its underlying mechanism of action by which it exerts a beneficial role in dermatological disease *in vivo*. In this study, we examined the immunomodulatory effects of *P. linteus* on experimental atopic dermatitis (AD) and elucidated its action mechanism.

**Methods:**

The immunomodulatory effect of total extract of *P. linteus* on IgE production by human myeloma U266B1 cells was measured by ELISA. To further identify the effective components, *P. linteus* was fractionated into methanol soluble, water soluble and boiling water soluble extracts. Each extract was treated to U266B1 cells and primary B cells to compare their inhibitory effects on IgE secretion. To test the *in vivo* efficacy, experimental atopic dermatitis (AD) was established by alternative treatment of DNCB and house dust mite extract into BALB/c mice. Water soluble extract of *P. linteus* (WA) or ceramide as a positive control were topically applied to ears of atopic mouse every day for 2 weeks and progression of the disease was estimated by the following criteria: (a) ear thickness, clinical score, (b) serum total IgE, IgG and mite specific IgE level by ELSIA, (c) histological examination of ear tissue by H&E staining and (d) cytokine profile of total ear cells and CD4^+^ T cells by real time PCR and ELSIA.

**Results:**

Treatment of total extracts of *P. linteus* to U266B1 inhibited IgE secretion. Among the diverse extracts of *P. linteus*, water soluble extract of *P. linteus* (WA) significantly reduced the IgE production in primary B cells and B cell line U266B1. Moreover, treatment of WA reduced AD symptoms such as ear swelling, erythema, and dryness and decreased recruitment of lymphocyte into the inflamed site. Interestingly WA treatment significantly reduced IgE level without affecting IgG levels and also down-regulated the levels of pathogenic cytokines (IL-4, IL-13, IL-12 and IFN-γ) and chemokines (CCL17 and CCL22) involved in AD development.

**Conclusions:**

Our study indicates that protective effect of water soluble extract of *P. linteus* in atopic dermatitis is mediated by inhibiting IgE production and expression of AD associated pathogenic cytokines as well as chemokines, suggesting the beneficial effect of *P. linteus* to modulate allergic skin disease.

## Background

Atopic dermatitis (AD) is a chronic inflammatory skin disease characterized by pruritic and eczematous skin lesions. The incidence of AD has rapidly increased especially in the industrialized countries and 10-20% of children in the world suffer from this disease [1]. AD is caused by complex pathogenic factors including genetic susceptibility, environment trigger, skin barrier dysfunction, bacterial infection and immune dysregulation [2]. AD is a complex immune reaction mediated by both T helper 1 (Th1) and Th2 immune responses. Th2 type cytokines including IL-4, IL-5 and IL-13 play important role in the development of AD by increasing the levels of serum IgE and blood eosinophils in AD patients [3]. Th1 type IFN-γ is also involved in the maintenance of chronic stage of AD by elevating the expression of CCL17 (TARC) and CCL22 (MDC) that are involved in the recruitment of effector T cells to the inflamed site [4]. IFN-γ increases the sensitivity of Fas-mediated apoptosis of keratinocytes, which is considered to be a key pathogenic event in eczematous dermatitis [5]. This immunological complexity associated with Th1/Th2 immune dysregulation makes it hard to properly modulate the AD symptoms. Although topical steroid therapy is widely used for the treatment of AD patients, diverse side effects limits its application. Development of new treatment methods is being initiated with herbal medicine with similar effectiveness but less side effects [6].

*Phellinus linteus* (*P. linteus*) has been known for its anti-cancer activity [7,8]. Recently, several reports suggested the anti-inflammatory and anti-allergic properties of this mushroom. N-butanol fraction of *P. linteus* inhibited croton-oil induced mouse edema in a dose dependent manner [9]. *P. linteus* extract reduces IgE production by increasing increased IFN-γ production [10]. Furthermore, boiling water fraction from mycelium of *P. linteus* inhibited mouse triphasic cutaneous reaction [11]. However, still the exact therapeutic effects of *P. linteus* and its underlying mechanisms in atopic dermatitis are unclear.

In the present study, we examined the therapeutic effects of cultured mycelium of *P. linteus* on the development of experimental AD in mice. Water soluble extract of *P. linteus* (WA) suppressed the IgE production by primary B cells and B cell line U266B1. Topical application of water soluble extract of *P. linteus* (WA) inhibited AD development by down-regulation of AD associated pathogenic cytokines and chemokines and by inhibition of lymphocytes infiltration into the inflamed skin region.

## Methods

### Animals

Female BALB/cCrSlc mice (6–8 weeks) were purchased from SLC Inc. (Hamamatsu, Japan). Mice were housed in specific pathogen-free barrier facilities and used in accordance with protocols approved by the Animal Care and Ethics Committees of the Gwangju Institute of Science and Technology (GIST).

### Fractionation and treatment of *Phellinus linteus*

**Figure 1:**
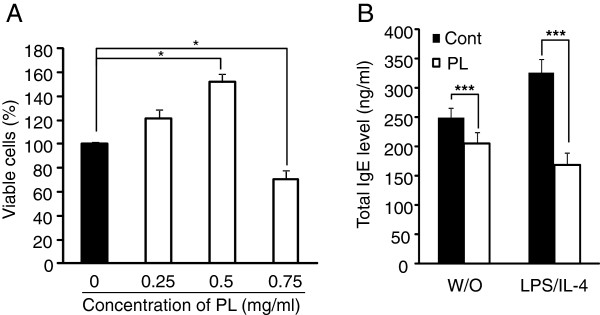
** Treatment of *P. linteus* inhibits IgE production in B cell line.** (**A**) Various concentrations of *P. linteus* extract were treated to U266B1 cell lines for 72 hrs and cell viability was measured by MTT assay. (**B**) U266B1 cells were stimulated with *P. linteus* (0.5 mg/ml) in the presence or absence of LPS (10 μg/ml), IL-4 (5 ng/ml) for 72 hrs and IgE levels in the culture supernatants were determined by ELISA. Error bars indicated S.D. One (*), two (**) and three asterisk (***) indicates P < 0.05, P < 0.005, P < 0.001, respectively. Data are representative of three independent experiments.

**Figure 2:**
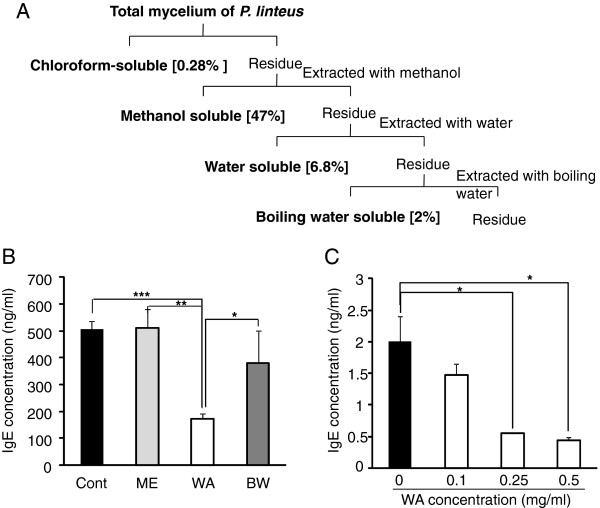
** Water soluble extract of *P. linteus* (WA) treatment reduces IgE secretion by B cells.** (**A**) Dried mycelium of *P. linteus* was extracted sequentially with chloroform, methanol, water and boiling water and then the fractions were freeze-dried resulting in a powdered extract. The yield of each fraction was indicated in the bracket. (**B**) The effect of each extract of *P. linteus*, ME (methanol soluble fraction), WA (water soluble fraction), BW (boiling water soluble fraction) on the secretion of IgE from U266B1 cell line was measured by ELISA. (**C**) B220+ B cells were isolated from lymph nodes and spleen of BALB/c mice. These primary B cells were stimulated with LPS (10 μg/ml), IL-4 (5 ng/ml) and various concentrations of WA for 72 hrs and IgE level was measured by ELISA. Error bars indicated S.D. One (*), two (**) and three asterisk (***) indicates P < 0.05, P < 0.005, P < 0.001, respectively. Data are representative of three independent experiments.

Mycelium of *P. linteus* was kindly provided by Derma Medico Co. (Seoul, Korea). Briefly, mycelium culture was carried out in a medium containing 42.5% sucrose, 0.5% yeast extract and 0.1% MgCl_2_ in distilled water, pH 7.0 in a 300 mL flask and incubated at 25°C for 6 days then separated by filter paper (Whatman®) and freeze dried. Ground dried mycelium of *P. linteus* dissolved in PBS was used to examine the effect of total *P. linteus* extracts (Figure 1). Constituents of the dried mycelium were extracted sequentially with chloroform, methanol, water and boiling water (Figure 2A). The extract was filtered and the supernatant was concentrated with a rotary evaporator and then freeze-dried resulting in a powder extract. Endotoxin level of each fraction measured by using Limulus Amebocyte Lysate kit (CAMBREX, East Rutherford, NJ, USA) was less than 50 EU/mg. The water soluble extract was dissolved in PBS (50 mg/ml) and 20 μl of water soluble extract was painted to the surface of both ear lobes every day during the induction period. The procedure and the yield of each fraction are summarized in Figure 2A.

### WST-1 assay

Cytotoxicity of *P. linteus* extract was conducted using EZ-Cytox cell viability assay kit (Daeil Lab Service Co, Seoul, Korea) by following manufacture’s protocol. Briefly, 5 × 10^3^ cells/well were dispensed in a 96 well plates and incubated for 24 hrs. Various concentrations of *P. linteus* extract were treated to the cells for 72 hrs and then incubated with 10 μl of reagent for 1 hr. Using the microplate reader, the absorbance was measured at 420–480 nm. Data was presented by relative growth inhibition compared to PBS-treated cells.

### Measurement of IgE secretion from U266B1 cells

Human U266B1 multiple myeloma cells (ATCC TIB-196™^TM^, American Type Culture Collection, USA) were cultured at 37°C under 5% CO_2_ incubator. RPMI 1640 medium supplemented with 10% FCS, 1 mM sodium pyuvate, 2 mM L-glutamine, 100 U penicillin and 50 μg/ml streptomycin was used as a culture medium. Cells (1 × 10^6^ cells/well) were stimulated with LPS (10 μg/ml), IL-4 (5 ng/ml) and *P. linteus* extract (0.5 mg/ml) for 72 hrs. The supernatants were harvested for IgE assay by Sandwich ELISA (BETHYL, Montgomery, TX, USA).

### Isolation of B220^+^ B cells and measurement of IgE production

Draining lymph nodes (superficial cervical, axillary, branchial lymph node) and spleen from normal BALB/c mice are grinded using cell strainer (BD Biosciences, San Diego, CA, USA), then B cells were isolated with B220^+^ B cell isolation bead (Miltenyi Biotech, Germany) and columns (Miltenyi Biotech, Germany) by following the manufacture’s protocol. To measure the secreted IgE level, primary B cells were stimulated with LPS (10 μg/ml), IL-4 (5 ng/ml) and various concentration of WA for 72 hrs then IgE concentration in the soup was measured by ELISA (BD Biosciences, San Diego, CA, USA).

### Induction of atopic dermatitis in the mouse ear

For the induction of atopic dermatitis, the surfaces of both ear lobes of mice were stripped with surgical tape (Nichiban, Japan). After stripping, 20 μl of 1% 2, 4-dinitrochlorobenzene (DNCB)(Sigma Aldrich, St Louis, MO, USA) dissolved in acetone/olive oil solution (acetone : olive oil = 1:3) was painted on each ear. After 3 days, 20 μl of mite extract (10 mg/ml, Dermatophagoides farinae, GREER source materials, Lenoir, NC, USA) dissolved in PBS containing 0.5% tween 20 was re-painted. Challenge of DNCB and mite extract was repeated once a week alternatively until 4 weeks. After 2 weeks of AD induction, mice were divided into 3 groups that have similar serum IgE levels. Then AD mice were treated daily with PBS (Cont), 1 mg/each ear of water soluble extract of *P. linteus* (WA) or 15 μg/each ear of ceramide (Cera) (C2-ceramice, Cayman chemical, Ann Arbor, Michigan, USA) until end of 4 weeks induction. Only tape stripping and PBS-painted group was used as a control (W/O).

### Measurement of ear swelling and clinical score

Ear thickness was measured 24 hrs after application of DNCB or mite extract with a dial thickness gauge (Kori Seiki MFG, Co., LTD., Japan). Mouse with representative clinical score of each group was photographed to show the clinical symptoms. Clinical symptoms of each mouse were evaluated as previously described [12]. Briefly, erythema, edema, excoriation and dryness on the ear surface was scored as 0 (not visible), 1 (mild), 2 (moderate), 3 (severe). Scoring was performed by three independent observers, and the final score was taken as an average for each group.

### Histological evaluation

Excised ears of each group were fixed in 4% paraformaldehyde for 16 hrs and embedded in paraffin. Then, 6 μm sections were stained with hematoxylin (Sigma Aldrich, St Louis, MO, USA) and eosin (H&E) (Sigma Aldrich, St Louis, MO, USA). Infiltrated lymphocytes, thickening of the epidermis and fibrosis in the dermis were observed by microscope.

### mRNA isolation, cDNA synthesis, quantitative RT-PCR

The changes in the levels of cytokine and chemokine mRNA expression in ears were determined by quantitative RT-PCR (qRT-PCR). Total RNA was isolated from ears of each group with TRIZol reagent (Molecular Research center, Cincinnati, OH, USA) according to manufacturer’s protocol. Reverse transcription was performed with reverse transcriptase primed by oligo (dT) primer (Promega, Madison, WI, USA). The synthesized cDNA were amplified by real-time PCR with specific primers: murine L32 (Forward 5′-GCC CAA GAT CGT CAA AAA GA-3′ and Reverse 5′-ATT GTG GAC CAG GAA CTT GC-3′), IL-4 (Forward 5′-ACA GGA GAA GGG ACG CCA T-3′ and Reverse 5′-GAA GCC GTA CAG ACG AGC TCA-3′), IL-5 (Forward 5′-AGC ACA GTG GTG AAA GAG AC-3′ and Reverse 5′-TCC AAT GCA TAG CTG GTG ATT T-3′), IL-10 (Forward 5′-ATA ACT GCA CCC ACT TCC CA-3′ and Reverse 5′-TCA TTT CCG ATA AGG CTT GG-3′), IL-12 (5′-GGA AGA CGG CAG CAG AAT A-3′ and Reverse 5′-AAC TTG AGG GAG AAG TAG GAA TGG-3′), IL-13 (Forward 5′-GCA ACA TCA CAC AGG ACC AGA-3′ and Reverse 5′-GTC AGG GAA TCC AGG GCT AC-3′), IFN-γ (Forward 5′-TCA AGT GGC ATA GAT GTG GAA GAA-3′ and Reverse 5′-TGG CTC TGC AGG ATT TTC ATG-3′), TNF-α (Forward 5′-CAT CTT CTC AAA ATT CGA GTG ACA A-3′), CCL17 (Forward 5′-CAT GAG GTC ACT TCA GAT GCT G-3′ and Reverse 5′-CCT GGA ACA CTC CAC TGA GG-3′), CCL22 (Forward 5′-AGG TCC CTA TGG TGC CAA TGT-3′ and Reverse 5′-CGG CAG GAT TTT GAG GTC CA-3′), Eotaxin (Forward 5′-TGA GAG GCT GAG ATC CAA-3 and Reverse 5′-CTG GGA GGT GAA GGA AGT-3′), CCL20 (Forward 5′-TGC TCT TCC T TG CTT TGG CAT GGG TA-3′ and Reverse 5′-TCT GTG CAG TGA TGT GCA GGT GAA GC-3′), CCR4 (Forward 5′-TCT ACA GCG GCA TCT TCT TCA T-3′ and Reverse 5′-CAG TAC GTG TGG TTG TGC TCT G-3′). The data was normalized using the expression levels of L32. Relative expression level of the each gene in the experimental group was compared with that of the control group.

### Measurement of immunoglobulin levels

Bloods were obtained from each treatment group at 2 weeks and 4 weeks of AD induction. Total IgE levels in the serum were measured using sandwich enzyme-linked immunosorbent assay (ELISA) kit (BD Biosciences, San Diego, CA, USA) by following the manufacturer’s protocol. For detection of mite specific IgE, mite extract (100 μl of 10 μg/ml/well) was coated in 96 well plates remaining procedures were followed according to the manufacture’s protocol. Antigen specific IgE levels were indicated by O.D value. Mean absorbance of antigen coated well minus mean absorbance of non-coated well was used as the O.D value of the mite specific IgE levels. For the detection of total IgG level, serum was analyzed with mouse IgG ELISA kit (BETHYL, Montgomery, TX, USA) by following the manufacturer’s protocol.

### Isolation of ear total cells and CD4^+^ T cells

Ears were removed from each treatment group, cut into three pieces and washed with RPMI medium and gently stirred in Erlenmeyer flasks containing 25 ml of 1.0 mM EDTA in 5% FBS for 20 mins at room temperature. Then ear segments were minced, transferred into 50 ml centrifuge tube containing 15 ml for RPMI without serum and vigorously shaken for 15 seconds three times. And the tissues were transferred into T flasks containing 10 ml of 0.5 mg/ml of Collagenase type V (Sigma Aldrich, St Louis, MO, USA), incubated for 1 hr at 37°C shaking incubator. After incubation, the soup containing ear total cells were centrifuged and washed with ice-cold PBS. Among total cells, some cells were analyzed for cytokines profile and then CD4^+^ T cells were isolated with CD4^+^ T cell isolation bead (Miltenyi Biotech, Germany) and columns (Miltenyi Biotech, Germany) according to the manufacture’s protocol.

### Cell culture and stimulation

The isolated primary cells were cultured in T cell media containing DMEM (Invitrogen, Carlsbad, CA, USA) supplemented with 10% FBS (Hyclone, USA), 3 mM L-glutamine (Sigma Aldrich, St Louis, MO, USA), 10 mM HEPES (Sigma Aldrich, St Louis, MO, USA), 100 U/ml penicillin (Sigma Aldrich, St Louis, MO, USA) and 100 U/ml streptomycin (Sigma Aldrich, St Louis, MO, USA) and 0.05 mM 2-beta-mercaptoethanol (Sigma Aldrich, St Louis, MO, USA). For cytokine profile analysis, cells were stimulated with PMA (0.5 μg/ml) and ionomycin (1 μM) for 4 hrs.

### Statistical analysis

A relative level of test group was compared with control value set at 1 or 100%. For statistical analysis, a two-tailed Student’s t-test was used to calculate the statistical significance of the experimental data. The level of significance was set at *P < 0.05, **P < 0.005 and ***P < 0.001. Significance was only indicated when appropriate.

## Results

### *P. linteus* extract reduces IgE production in B cell line

To find out the optimal concentration of *P. linteus* (PL) extract for *in vitro* efficacy test, cytotoxicity of *P. linteus* extracts was tested. Human U266B1 multiple myeloma cells were cultured in the presence of various concentrations of *P. linteus* extract for 72 hrs then relative viable cells were measured by WST-1 assay (Figure 1A). Treatment of low concentration of *P. linteus* (< 0.5 mg/ml) was not toxic to cells rather enhanced viable cell numbers were observed due to the cell proliferation during the culture periods. However, at 0.75 mg/ml viable cells were suddenly decreased (Figure 1A). So based on this result, 0.5 mg/ml of PL extract was applied to all the *in vitro* experiments. Next we tested the effect of total extracts of *P. linteus* on IgE production by U266B1 cells that established from the peripheral blood of myeloma patients and secrete highly levels of IgE [13]. Cells were stimulated with LPS (10 μg/ml) and IL-4 (5 ng/ml) in the presence of *P. linteus* extract or PBS as a control for 72 hrs and then the levels of IgE production in the culture supernatant were measured by ELISA. Treatment of *P. linteus* extract significantly reduced IgE production compared to control treatment in both without (W/O) and LPS/IL-4 stimulation condition and inhibitory effect by *P. linteus* was enhanced upon stimulation (Figure 1B).

### Water soluble extract of *P. linteus* (WA) inhibits IgE production

To further identify the potent inhibitory fraction on IgE production, the mycelia of *P. linteus* were extracted with methanol (ME), water (WA) and boiling water (BW) by the sequential fractionation method as described in Figure 2A. Each extract was adjusted to have the same concentration (0.5 mg/ml) and added to the culture media of U266B1 cells. Cells were stimulated with LPS (10 μg/ml) and IL-4 (5 ng/ml) in the presence of indicated extract of *P. linteus* or PBS (Cont) for 72 hrs and then IgE level in the culture supernatants were measured by ELISA. Among the three extracts, WA significantly suppressed IgE secretion compared to the control (Figure 2B) while methanol (ME) or boiling water extract (BW) failed to do. To further check whether WA can also effectively reduce IgE production by primary B cells, WA was applied to mouse primary B cells. Indeed, WA treatment significantly decreased IgE production in a dose dependent manner (Figure 2C). These results indicate that water soluble extract of *P. linteus* (WA) can inhibit the IgE secretion by activated primary B cells.

### Topical application of water soluble extract of *P. linteus* (WA) alleviates the symptoms of experimental atopic dermatitis

**Figure 3:**
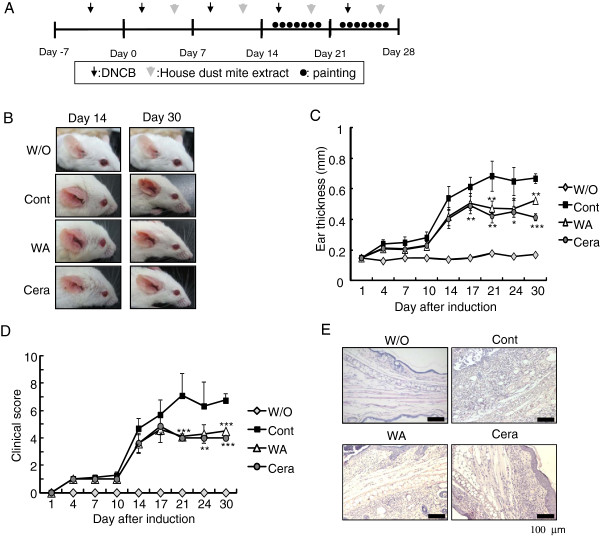
** Topical application of water soluble extract of *P. linteus* improves the symptoms of experimental atopic dermatitis.** (**A**) Experimental AD was induced on the both ears of BALB/c mice by alternative painting of DNCB (0.2 mg/each ear) and mite extract (0.2 mg/each ear) for 2 weeks. After 2 weeks of AD induction, control PBS (Cont) or water soluble extract of *P. linteus* (WA, 1 mg/each ear) or ceramide (Cera, 15 μg/each ear) were painted daily for 2 weeks while continuously inducing experimental atopic dermatitis. (**B**) Representative mice of each treatment group at day 14 and day 30 are shown. During AD induction period, ear thickness (**C**) and clinical score (**D**) was measured 24 hrs after application of DNCB or mite extract. (E) Infiltrated lymphocytes into ears were observed by H&E staining. Error bars indicated S.D. One (*), two (**) and three asterisk (***) indicates P < 0.05, P < 0.005, P < 0.001, respectively. Data are representative of three independent experiments. Without AD induction (normal health mice) is indicated as “W/O”.

Then to evaluate the immunomodulatory function of *P. linteus in vivo*, water soluble extract of *P. linteus* (WA) was topically applied to the ears of atopic dermatitis (AD) induced mice. Experimental AD was induced on the both ears of BALB/c mice by alternative painting of DNCB and mite extract for 2 weeks [14,15]. After 2 weeks of induction, mice were divided into 3 groups and daily painted with PBS (Cont), water soluble extract of *P. linteus* (WA) or ceramide (Cera) to both ears for 2 weeks. To compare the therapeutic effects of WA, ceramide was employed as a positive control [16-21]. Painting of WA significantly reduced the AD symptoms including erythema, horny substance, dryness, and scaling (Figure 3B) by reducing ear thickness (Figure 3C) and clinical score (Figure 3D) compared with the PBS-treated control group. Interestingly, the therapeutic efficacy of WA was comparable to the ceramide-treated group. To further confirm the visual evaluation of AD symptoms, histological analysis on atopic ears was performed. Excised ears from each group were stained with hematoxylin and eosin and infiltrated lymphocytes, thickening of the epidermis and fibrosis in the dermis were observed by microscope. Indeed, compared with PBS-treated control group, WA treated group showed significantly reduced number of infiltrated immune cells such as lymphocytes, eosinophils, mast cells and thickness of epidermis (Figure 3E). Collectively, these data suggest that water soluble extract of *P. linteus* (WA) has protective effect on the progression of murine atopic dermatitis. 

### Water soluble extract of *P. linteus* (WA) reduces serum IgE levels

**Figure 4:**
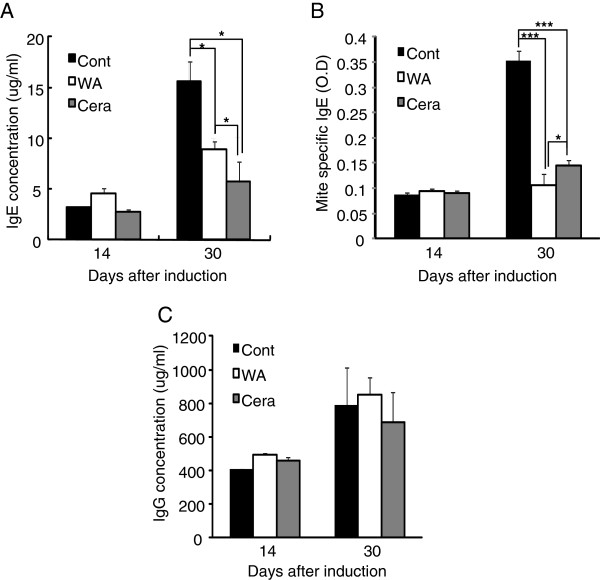
** Water soluble extract of *P. linteus* (WA) reduces IgE levels in AD induced mice.** After 2 and 4 weeks of AD induction, sera were obtained from each group, and the levels of total IgE (**A**), antigen (mite)-specific IgE (**B**) and total IgG (**C**) were measured by ELISA. Error bars indicated S.D. One (*), two (**) and three asterisk (***) indicates P < 0.05, P < 0.005, P < 0.001, respectively. Data are representative of three independent experiments.

Increased IgE level is the hall mark of atopic dermatitis progression. To further test whether suppression of AD progression by the treatment of WA is also associated with changes in IgE levels, serum IgE levels were measured from each treatment group. After 14 and 30 days of AD induction, serum was obtained from each treatment group, and total IgE and mite specific IgE levels were measured by ELISA. In control group, IgE levels were increased in an induction time dependent manner. However, topical application of WA significantly reduced serum IgE levels which were comparable to that of ceramide treated mice (Figure 4A) without affecting serum total IgG levels (Figure 4C). In addition, treatment of WA significantly (p < 0.005) reduced the mite specific serum IgE levels even better than ceramide treated group (Figure 4B). These data indicate that therapeutic effect of water soluble extract of *P. linteus* (WA) in AD progression is linked with down-regulation of serum IgE levels.

### Water soluble extract of *P. linteus* (WA) reduces the expression of pathogenic cytokines and chemokines

**Figure 5:**
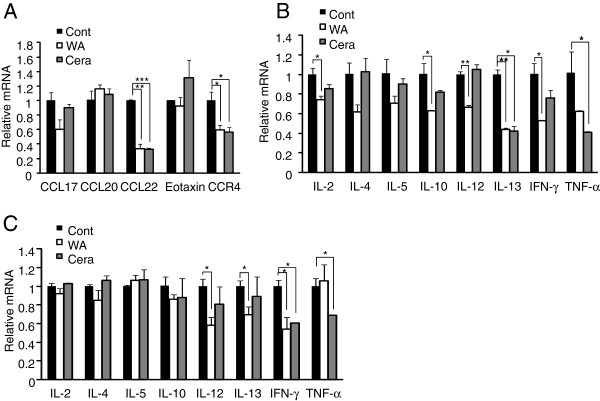
** Water soluble extract of *P. linteus* (WA) inhibits the expression of pathogenic cytokines and chemokines in ear.** After final treatment, mice of each group were sacrificed and ears were removed. (**A, B**) Total ear cells were stimulated with PMA (0.5 μg/ml) and ionomycin (1 μM) for 4 hrs, then mRNA expression of chemokines (**A**) and cytokines (**B**) were determined by quantitative RT-PCR. (C) CD4+ T cells isolated from ear total cells were stimulated and cytokine levels were measured by quantitative RT-PCR. The mRNA expression levels in each group were normalized with L32 (house- keeping gene) and then fold induction of each target gene were compared to Cont (PBS) group. Error bars indicated S.D. One (*), Two (**) and three asterisk (***) indicates P < 0.05, P < 0.005, P < 0.001, respectively. Data are representative of three independent experiments.

Together with increased IgE levels, T helper (Th1), Th2 type cytokines and chemokines also mediate progression of atopic dermatitis by recruiting lymphocytes, mast cells and eosinophils to the atopic skins. We further examined whether suppression of AD progression by the treatment of WA is mediated by the changes in the levels of AD associated cytokines and chemokines in total ear cells or ear residual CD4^+^ T cells. Total ear cells isolated from each group were stimulated with PMA (0.5 μg/ml) and ionomycin (1 μM) for 4 hrs and the expressions of cytokines and chemokines were compared between the treatment groups. In line with the histological analysis, WA treatment significantly decreased CCL22 and CCR4 expression levels (Figure 5A). Ceramide treatment also significantly reduced CCL22 and CCR4 expression. In addition, treatment of WA significantly decreased the expression of pathogenic cytokines [22] including IL-2, IL-10, IL-12, IL-13 and IFN-γ and ceramide treatment significantly reduced IL-13 and TNF-α expression (Figure 5B). Next, to further define the effects of WA treatment on effector T cells, the expression levels of cytokines in ear residual CD4^+^ T cells were measured by quantitative RT-PCR. WA or ceramide treatment significantly down-regulated pathogenic cytokine expression such as IL-12, IL-13 and IFN-γ compared with PBS (Cont) (Figure 5C). These data indicate that protective effect of WA in AD progression is mediated by down-regulation of pathogenic chemokines and cytokines in the inflamed tissues, resulting in significant reduction of infiltrated pathogenic immune cells. 

## Discussion

In this study, we identified a novel function of *P. linteus* as a potent therapeutic modulator for atopic dermatitis (AD) and elucidated the underlying action mechanisms of it in alleviation of AD symptoms. Treatment of water soluble extract of *P. linteus* (WA) significantly reduced serum IgE and the levels of AD related pathogenic cytokines and chemokines.

Atopic dermatitis is thought to be a typical Th2 type immune disorder which shows elevated serum IgE level and increment of Th2 type cytokines such as IL-4, IL-5 and IL-13 [23,24]. Th1 type response also plays key role in pathogenesis and maintenance of AD [23]. Clinical trials have been performed to modulate Th2 and Th1 type responses as well as chemokines levels. General immune suppressants have been used to treat AD, which cause numerous side effects with short period of efficacy. Recently herbal medicines have become a major part of CAMs (Complementary and Alternative Medicines) for treatment various kinds of diseases including cancer, allergy and diabetes [25]. These plant derived products have been used as drugs and food additives for a long time. However, scientific evidence is required for the further development of herbal medicines and their clinical application to treat diverse diseases. *P. linteus* is a member of Hymenochaetaceae, which has been used as a traditional medicine in oriental countries for the treatment of various diseases such as gastroenteric disorder, inflammation, tumors and lymphopathic disease [26,27]. Its pharmacological activities, especially anti-tumor and anti-inflammatory activities, have been documented [28-30]. However no information is available on the effect of mycelium of *P. linteus* in modulation of allergic skin disorders such as atopic dermatitis.

In this study we aimed to test and identify effective fraction of the mycelium of *P. linteus* in modulation of atopic dermatitis. Since *P. linteus* has limited production in nature, we used mycelium culture of *P. linteus* which can be easily available in large quantities. To elucidate whether *P. linteus* has therapeutic potential for atopic dermatitis, we first tested inhibitory effect on IgE production by B cells since serum IgE levels were considered as an authentic marker of AD. Indeed, 70 ~ 80% of AD patients showed significantly increased serum IgE level compared to non-AD patients. After determining the non-cytotoxic concentration we applied total extract of *P. linteus* into IgE producing U266B1 B cell lines and found that the extract significantly inhibited IgE production under the LPS and IL-4 stimulation which is the well known IgE class switching condition [13,31] as well as W/O stimulation (Figure 1B). To identify potent inhibitory fraction we further fractionated *P. linteus* by diverse solvents including chloroform, methanol, water and boiling water (Figure 2A). Among the extracts, water soluble extract of *P. linteus* (WA) showed the most potent inhibitory activity to block IgE production in both U266B1 cell line and primary B cells (Figure 2B). In accord with *in vitro* result, among the three fractions only water soluble extract of *P. linteus* (WA) significantly decreased total serum IgE levels from AD induced mice (data not shown). Based on these results, we investigated the therapeutic effect of soluble extract of *P. linteus* (WA) in ongoing AD model. We also used ceramide [21] as a positive control to elucidate remedial value of soluble extract of *P. linteus* (WA). It has been reported that ceramide content is decreased in the skin of AD patients [32] and ceramide content in the stratum corneum showed a correlation with skin barrier function in AD patients. Indeed, topical application of ceramide derivatives suppressed AD-like skin lesions in NC/Nga mice by inhibiting infiltration of leukocytes and mast cells and reduced IL-4, TNF-α expression from ear cells [33]. As expected, topical application of ceramide significantly reduced AD symptoms, ear thickness and increment of clinical scores compared to PBS treatment (Figure 3). Interestingly, treatment of water soluble extract of *P. linteus* (WA) showed comparable therapeutic effects with ceramide in reducing AD symptoms such as ear thickness and clinical score (Figure 3). Histological analysis of ear tissues further indicated that treatment water soluble extract of *P. linteus* (WA) significantly decreased tissue infiltration of lymphocytes and granulocytes compared with ear tissues from PBS treated control mice (Figure 3E). In addition, treatment of water soluble extract of *P. linteus* (WA) significantly reduced total IgE levels (Figure 4A) as well as in antigen (mite)-specific IgE levels (Figure 4B) without affecting total IgG levels (Figure 4C). Interestingly, water soluble extract of *P. linteus* (WA) was more potent than ceramide in reducing mite-specific IgE levels. These data indicated that *P. linteus* can suppress allergic responses in an allergen specific manner; consequently, this could be one of the merits of *P. linteus* for the treatment of atopic dermatitis.

AD pathogenesis is associated with recruitment of lymphocyte, granulocyte to the inflammatory skin region. During this multiple step process of leukocyte trafficking and migration, chemokine ligand-receptor interactions are considered as crucial factors. Several chemokines have association with AD phenotype and among them, CCL17, CCL22, CCR4 have pivotal roles in migration of pathogenic immune cells (mainly CD4^+^ T cells) to the site of inflammation [34]. Interestingly, treatment of water soluble extract of *P. linteus* (WA) significantly decreased the expression of CCL22 and CCR4 (Figure 5A). Since keratinocyte, epithelial cells and dendritic cells are major source of these chemokines, suppression of chemokine expression by the treatment of water soluble extract of *P. linteus* (WA) could lead to lowering of infiltration of pathogenic T cells at the site of inflammation (Figure 3E) [35-38]. Decreased expression of CCR4 also indicates that water soluble extract of *P. linteus* (WA) could inhibit T cell differentiation into Th2 cells since CCR4 is mainly expressed on the surface of Th2 cells in AD patients. Inhibitory effect of water soluble extract of *P. linteus* (WA) on the chemokine expression was comparable with that of ceramide (Figure 5A). Like chemokines, cytokines are also crucial pathogenic factors in AD pathogenesis. Both Th1 and Th2 type cytokines contribute to the pathogenesis of AD and their expression pattern is not mutually exclusive [39]. IL-4, IL-5 and IL-13 are typical Th2 type cytokines which stimulates Th2 differentiation and IgE production by B cells. IL-12 and IFN-γ are typical Th1 type cytokines that induce differentiation and maturation of T cells into Th1 type cells. Development of AD is induced by Th2 type response, while the chronic inflammatory responses is dominantly mediated by Th1 type reactions [40]. In addition, house dust mite reactive T cells produce both Th1 and Th2 type cytokines which strongly supports this concept [41]. Therefore, for the treatment of AD, both Th1 and Th2 types of immune responses should be considered as therapeutic targets. To elucidate the underlying mechanism of water soluble extract of *P. linteus* (WA), we measured mRNA expression levels of AD-related pathogenic cytokines from ear tissues and ear residual CD4^+^ T cells (Figure 5B and 5C). In ear tissues, topical application of water soluble extract of *P. linteus* (WA) significantly reduced not only Th2 cytokines (IL-10 and IL-13) but also Th1 cytokines (IL-12 and IFN-γ) (Figure 5B). IL-13 is known as the major inducer of Th2 generation in the cutaneous microenvironment, independent of IL-4 [42]. IL-12 may have critical roles to terminate Th2 cytokine expression but it also initiates expression of Th1 cytokine IFN- γ which induces CCL17 (TARC) and CCL22 (MDC) from keratinocyte and epithelial cells [43-45]. Interestingly, treatment of water soluble extract of *P. linteus* (WA) significantly decreased expression of MHCII, B7.1 and B7.2 in ear tissues of AD mice compared to PBS treated mice (data not shown). Hence, treatment with water soluble extract of *P. linteus* (WA) may suppress activation of antigen presenting cells including dendritic cells, macrophage, keratinocyte and epithelial cells at the site of inflammation, which modulates activation and differentiation of CD4^+^ T cells into Th1 or Th2 type. Interestingly, treatment of water soluble extract of *P. linteus* (WA) more significantly down-regulated the expression levels of IL-2, IL-10, IL-12, and IFN-γ from ear cells compared to ceramide treatment (Figure 5B). Indeed, treatment with water soluble extract of *P. linteus* (WA) significantly decreased expression level of pathogenic cytokines including IL-12, IL-13 and IFN- γ in CD4+ T cells of atopic regions and the inhibitory effect was more efficient than ceramide treatment (Figure 5C). In addition, consistent with PMA/ionomycin stimulation, under the antigen specific stimulation by mite extract, WA also significantly decreased the expression levels of pathogenic cytokines (IL-4, IL-13 and IFN-γ) and chemokines (CCL22) and this effect was more effective than ceramide treatment (Additional file 1: Figure S 1). Among the subfractions, water soluble extract of *P. linteus* (WA) was the most effective in modulating AD-associated inflammatory responses, implying that *P. linteus* may contain active anti-inflammatory component(s) with relatively hydrophilic characters. Further characterization of active compound will lead to develop a potent AD modulating agent. Topical treatment of ointment containing extract of *P. linteus* significantly decreased total serum IgE levels. Furthermore, not only topical treatment of WA, oral administration of WA also improved AD symptoms including reduction of IgE levels, pathogenic cytokine expression and immune cell infiltrations (data not shown). *P. linteus* is well known to exhibit anti-cancer effects through immuno-potentiating effects and also anti-inflammatory effects. Exact action mechanisms of anti-tumor and anti-inflammatory effect might be mediated by different active component. In addition, routes of treatments such as oral administration or topical application and target cells of *P. linteus* under the certain disease environment may mediate diverse effect of *P. linteus* on different immune disorders.

Collectively, topical application of water soluble extract of *P. linteus* (WA) may inhibit hyper-activation of tissue residual antigen presenting cells, which subsequently block the initiation of immune cascade from innate to adaptive immunity.

## Conclusions

Although beneficial effects of *P. linteus* in various inflammatory diseases have been noticed for a long time, the exact action mechanism was not clear. In this study we have shown that topical application of water soluble extract of mycelium of *P. linteus* (WA) inhibits the development of experimental AD by reducing the infiltration of leukocytes and granulocytes and by decreasing serum IgE levels. Thus our results indicate that water soluble extract of mycelium of *P. linteus* (WA) could be applicable as an effective complementary and alternative medicine to modulate atopic symptoms.

## Abbreviations

P.L: Total extract of *P. linteus*; WA: Water soluble extract of *P. linteus*; ME: Methanol extract of *P. linteus*; BW: Boiling water extract of *P. linteus*; Cera: Ceramide; Cont: Control; LPS: Lipopolysaccharide; PMA: Phorbol 12-myristate 13-acetate; CAM: Complementary and alternative medicine; AD: Atopic dermatitis; Th1 & Th2: T helper 1 and T helper 2; IL-2: Interleukin 2; IL-4: Interleukin 4; IL-5: Interleukin 5; IL-10: Interleukin 10; IL-12: Interleukin 12; IL-13: Interleukin 13; IFN-γ: Interferon gamma; TNF-α: Tumor necrosis factor alpha; DNCB: 2, 4-dinitrocholorobenzene; PBS: Phosphate buffer saline; CCL17: Chemokine (C-C motif) ligand 17; CCL20: Chemokine (C-C motif) ligand 20; CCL22: Chemokine (C-C motif) ligand 22; CCR4: C-C chemokine receptor type 4.

## Competing interests

The authors declare that they have no competing interests.

## Authors’ contributions

S-HI designed the research; J-SH, H-KK, J-EK and J-HR conducted research; J-SH and S-HI analyzed data; J-SH and S-HI wrote the paper; S-HI had primary responsibility for final content. All authors read and approved the final manuscript.

## Pre-publication history

The pre-publication history for this paper can be accessed here:

http://www.biomedcentral.com/1472-6882/12/159/prepub

## Supplementary Material

Additional file 1 ** Figure S1. **Water soluble extract of *P. linteus* (WA) inhibits the expression of pathogenic cytokines and chemokines under the antigen specific stimulation. AD-induced mice were sacrificed and ears were removed. Total ear cells were stimulated with WA (0.5 mg/ml) or ceramide (10 μg /ml) in the presence of mite extract (5 μg /ml) for 24 hrs, then mRNA expression of chemokines (A) and cytokines (B) were determined by quantitative RT-PCR. The mRNA expression levels of each sample were normalized with L32 (house- keeping gene) and then fold induction of each target gene were compared to PBS treated control. Error bars indicated S.D. One (*), Two (**) and three asterisk (***) indicates P < 0.05, P < 0.005, P < 0.001, respectively. Data are representative of three independent experiments. Click here for file

## References

[B1] Leung DYM Jain N Leo HL New concepts in the pathogenesis of atopic dermatitis Curr Opin Immunol 2003 15 6 634 638 10.1016/j.coi.2003.09.009 14630196

[B2] Leung DYM Boguniewicz M Howell MD Nomura I Hamid QA New insights into atopic dermatitis J Clin Invest 2004 113 5 651 657 14991059 10.1172/JCI21060 PMC351324

[B3] Homey B Steinhoff M Ruzicka T Leung DYM Cytokines and chemokines orchestrate atopic skin inflammation J Allergy Clin Immunol 2006 118 1 178 189 10.1016/j.jaci.2006.03.047 16815153

[B4] Pivarcsi H Chemokine networks in atopic dermatitis: traffic signals of disease Curr Allergy Asthma Rep 2005 5 4 284 290 10.1007/s11882-005-0068-y 15967069

[B5] Trautmann A Akdis M Kleemann D Altznauer F Simon H-U Graeve T Noll M Eva-B B Blaser K T cell-mediated Fas-induced keratinocyte apoptosis plays a key pathogenetic role in eczematous dermatitis J Clin Invest 2000 106 1 25 35 10.1172/JCI9199 10880045 PMC517909

[B6] Roland NJ Bhalla RK Earis J The Local Side Effects of Inhaled Corticosteroids* Chest 2004 126 1 213 219 10.1378/chest.126.1.213 15249465

[B7] Nakamura T Matsugo S Uzuka Y Matsuo S Kawagishi H Fractionation and Anti-tumor Activity of the Mycelia of Liquid-cultured Phellinus linteus Biosci Biotechnol Biochem 2004 68 4 868 872 10.1271/bbb.68.868 15118316

[B8] Han SB Lee CW Jeon YJ Hong ND Yoo ID Yang K-H Kim HM The inhibitory effect of polysaccharides isolated from Phellinus linteus on tumor growth and metastasis Immunopharmacology 1999 41 2 157 164 10.1016/S0162-3109(98)00063-0 10102797

[B9] Kim S-H Song Y-S Kim S-K Kim B-C Lim C-J Park E-H Anti-inflammatory and related pharmacological activities of the n-BuOH subfraction of mushroom Phellinus linteus J Ethnopharmacol 2004 93 1 141 146 10.1016/j.jep.2004.03.048 15182919

[B10] Lim BO Yamada K Cho B-G Jeon T Hwang S-G Park T Kang SA Park DK Comparative Study on the Modulation of IgE and Cytokine Production by Phellinus linteus Grown on Germinated Brown Rice, Phellinus Linteus and Germinated Brown Rice in Murine Splenocytes Biosci Biotechnol Biochem 2004 68 11 2391 2394 10.1271/bbb.68.2391 15564681

[B11] Inagaki N Shibata T Itoh T Suzuki T Tanaka H Nakamura T Akiyama Y Kawagishi H Nagai H Inhibition of IgE-dependent Mouse Triphasic Cutaneous Reaction by a Boiling Water Fraction Separated from Mycelium of Phellinus linteus Evidence-Based Complementary and Alternative Medicine 2005 2 3 369 374 10.1093/ecam/neh105 16136215 PMC1193546

[B12] Matsuoka H Maki N Yoshida S Arai M Wang J Oikawa Y Ikeda T Hirota N Nakagawa H Ishii A A mouse model of the atopic eczema/dermatitis syndrome by repeated application of a crude extract of house-dust mite Dermatophagoides farinae Allergy 2003 58 2 139 145 10.1034/j.1398-9995.2003.23790.x 12622745

[B13] Kim HM Kim HJ Park ST Inhibition of immunoglobulin E production by Poncirus trifoliata fruit extract J Ethnopharmacol 1999 66 3 283 288 10.1016/S0378-8741(99)00028-8 10473174

[B14] Kwon H-K Lee C-G So J-S Chae C-S Hwang J-S Sahoo A Nam JH Rhee JH Hwang K-C Im S-H Generation of regulatory dendritic cells and CD4 + Foxp3+ T cells by probiotics administration suppresses immune disorders Proc Natl Acad Sci 2010 107 5 2159 2164 10.1073/pnas.0904055107 20080669 PMC2836639

[B15] Choi E-J Lee S Kim H-H Singh TSK Choi JK Choi HG Suh WM Lee S-H Kim S-H Suppression of dust mite extract and 2,4-dinitrochlorobenzene-induced atopic dermatitis by the water extract of Lindera obtusiloba J Ethnopharmacol 2011 137 1 802 807 10.1016/j.jep.2011.06.043 21762765

[B16] Berardesca E Barbareschi M Veraldi S Pimpinelli N Evaluation of efficacy of a skin lipid mixture in patients with irritant contact dermatitis, allergic contact dermatitis or atopic dermatitis: a multicenter study Contact Dermatitis 2001 45 5 280 285 10.1034/j.1600-0536.2001.450505.x 11722487

[B17] Kucharekova M Schalkwijk J Van De Kerkhof PCM Van De Valk PGM Effect of a lipid-rich emollient containing ceramide 3 in experimentally induced skin barrier dysfunction Contact Dermatitis 2002 46 6 331 338 10.1034/j.1600-0536.2002.460603.x 12190621

[B18] Atanasković-Marković M Ćirković Veličković T Gavrović-Jankulović M Ivanovski P Nestorović B A case of selective IgE-mediated hypersensitivity to ceftibuten Allergy 2005 60 11 1454 1454 10.1111/j.1398-9995.2005.00912.x 16197484

[B19] Kimata H Improvement of Atopic Dermatitis and Reduction of Skin Allergic Responses by Oral Intake of Konjac Ceramide Pediatr Dermatol 2006 23 4 386 389 10.1111/j.1525-1470.2006.00268.x 16918640

[B20] Park E-J Kim B Eo H Park K Kim Y Lee HJ Son M Chang Y-S Cho S-H Kim S Control of IgE and selective TH1 and TH2 cytokines by PG102 isolated from Actinidia arguta J Allergy Clin Immunol 2005 116 5 1151 1157 10.1016/j.jaci.2005.07.024 16275390

[B21] Chamlin SL Kao J Frieden IJ Sheu MY Fowler AJ Fluhr JW Williams ML Elias PM Ceramide-dominant barrier repair lipids alleviate childhood atopic dermatitis: Changes in barrier function provide a sensitive indicator of disease activity J Am Acad Dermatol 2002 47 2 198 208 10.1067/mjd.2002.124617 12140465

[B22] Novak N Bieber T Leung DYM Immune mechanisms leading to atopic dermatitis J Allergy Clin Immunol 2003 112 6, Supplement 1 S128 S139 10.1016/j.jaci.2003.09.032 14657843

[B23] Chen L Martinez O Overbergh L Mathieu C Prabhakar BS Chan LS Early up-regulation of Th2 cytokines and late surge of Th1 cytokines in an atopic dermatitis model Clin Exp Immunol 2004 138 3 375 387 10.1111/j.1365-2249.2004.02649.x 15544612 PMC1809236

[B24] Neis MM Peters B Dreuw A Wenzel J Bieber T Mauch C Krieg T Stanzel S Heinrich PC Merk HF Enhanced expression levels of IL-31 correlate with IL-4 and IL-13 in atopic and allergic contact dermatitis J Allergy Clin Immunol 2006 118 4 930 937 10.1016/j.jaci.2006.07.015 17030248

[B25] Kraft K Complementary/Alternative Medicine in the context of prevention of disease and maintenance of health Prev Med 2009 49 2–3 88 92 19465045 10.1016/j.ypmed.2009.05.003

[B26] Choi YH Yan GH Chai OH Lim JM Sung SY Zhang X Kim J-H Choi SH Lee MS Han E-H Inhibition of Anaphylaxis-Like Reaction and Mast Cell Activation by Water Extract from the Fruiting Body of Phellinus linteus Biol Pharm Bull 2006 29 7 1360 1365 10.1248/bpb.29.1360 16819169

[B27] Kim G-Y Kim S-H Hwang S-Y Kim H-Y Park Y-M Park S-K Lee M-K Lee S-H Lee T-H Lee J-D Oral Administration of Proteoglycan Isolated from Phellinus linteus in the Prevention and Treatment of Collagen-Induced Arthritis in Mice Biol Pharm Bull 2003 26 6 823 831 10.1248/bpb.26.823 12808294

[B28] Kim B-C Jeon W-K Hong H-Y Jeon K-B Hahn J-H Kim Y-M Numazawa S Yosida T Park E-H Lim C-J The anti-inflammatory activity of Phellinus linteus (Berk. & M.A. Curt.) is mediated through the PKC[delta]/Nrf2/ARE signaling to up-regulation of heme oxygenase-1 J Ethnopharmacol 2007 113 2 240 247 10.1016/j.jep.2007.05.032 17644290

[B29] Kim G-Y Roh S-I Park S-K Ahn S-C Oh Y-H Lee J-D Park Y-M Alleviation of Experimental Septic Shock in Mice by Acidic Polysaccharide Isolated from the Medicinal Mushroom Phellinus linteus Biol Pharm Bull 2003 26 10 1418 1423 10.1248/bpb.26.1418 14519947

[B30] Kim HG Yoon DH Lee WH Han SK Shrestha B Kim CH Lim MH Chang W Lim S Choi S Phellinus linteus inhibits inflammatory mediators by suppressing redox-based NF-[kappa]B and MAPKs activation in lipopolysaccharide-induced RAW 264.7 macrophage J Ethnopharmacol 2007 114 3 307 315 10.1016/j.jep.2007.08.011 17936530

[B31] Mandler R Finkelman FD Levine AD Snapper CM IL-4 induction of IgE class switching by lipopolysaccharide-activated murine B cells occurs predominantly through sequential switching J Immunol 1993 150 2 407 418 8419474

[B32] Imokawa G Lipid abnormalities in atopic dermatitis J Am Acad Dermatol 2001 45 1 S29 S32 10.1067/mjd.2001.117020 11423869

[B33] Kang JS Yoon WK Youm J-K Jeong SK Park BD Han MH Lee H Moon E-Y Han S-B Lee CW Inhibition of atopic dermatitis-like skin lesions by topical application of a novel ceramide derivative, K6PC-9p, in NC/Nga mice Exp Dermatol 2008 17 11 958 964 10.1111/j.1600-0625.2008.00737.x 18721197

[B34] Shimada Y Takehara K Sato S Both Th2 and Th1 chemokines (TARC/CCL17, MDC/CCL22, and Mig/CXCL9) are elevated in sera from patients with atopic dermatitis J Dermatol Sci 2004 34 3 201 208 10.1016/j.jdermsci.2004.01.001 15113590

[B35] Nakazato J Fujiwara J Inoue M Kishida M Shinomiya N TARC(CCL17) Augments TNF-a Induced CTACK(CCL27) Production in Infantile Atopic Dermatitis J Allergy Clin Immunol 2007 119 1 S282

[B36] Hashimoto S Nakamura K Oyama N Kaneko F Tsunemi Y Saeki H Tamaki K Macrophage-derived chemokine (MDC)/CCL22 produced by monocyte derived dendritic cells reflects the disease activity in patients with atopic dermatitis J Dermatol Sci 2006 44 2 93 99 10.1016/j.jdermsci.2006.08.004 17008059

[B37] Maeda S Tsukui T Saze K-i Masuda K Ohno K Tsujimoto H Iwabuchi S Production of a monoclonal antibody to canine thymus and activation-regulated chemokine (TARC) and detection of TARC in lesional skin from dogs with atopic dermatitis Vet Immunol Immunopathol 2005 103 1–2 83 92 15626464 10.1016/j.vetimm.2004.08.021

[B38] Grewe M Bruijnzeel-Koomen CAFM Schif E Thepen T Langeveld-Wildschut AG Ruzicka T Krutmann J A role for Th1 and Th2 cells in the immunopathogenesis of atopic dermatitis Immunol Today 1998 19 8 359 361 10.1016/S0167-5699(98)01285-7 9709503

[B39] Ohmen JD Hanifin JM Nickoloff BJ Rea TH Wyzykowski R Kim J Jullien D McHugh T Nassif AS Chan SC Overexpression of IL-10 in atopic dermatitis. Contrasting cytokine patterns with delayed-type hypersensitivity reactions J Immunol 1995 154 4 1956 1963 7836775

[B40] Horikawa T Nakayama T Hikita I Yamada H Fujisawa R Bito T Harada S Fukunaga A Chantry D Gray PW IFN-gamma-inducible expression of thymus and activation-regulated chemokine/CCL17 and macrophage-derived chemokine/CCL22 in epidermal keratinocytes and their roles in atopic dermatitis Int Immunol 2002 14 7 767 773 10.1093/intimm/dxf044 12096036

[B41] Wierenga EA Snoek M Jansen HM Bos JD van Lier RA Kapsenberg ML Human atopen-specific types 1 and 2 T helper cell clones J Immunol 1991 147 9 2942 2949 1680923

[B42] Herrick CA Xu L McKenzie ANJ Tigelaar RE Bottomly K IL-13 Is Necessary, Not Simply Sufficient, for Epicutaneously Induced Th2 Responses to Soluble Protein Antigen J Immunol 2003 170 5 2488 2495 12594274 10.4049/jimmunol.170.5.2488

[B43] Jahnz-Rozyk K Targowski T Paluchowska E Owczarek W Kucharczyk A Serum thymus and activation-regulated chemokine, macrophage-derived chemokine and eotaxin as markers of severity of atopic dermatitis Allergy 2005 60 5 685 688 10.1111/j.1398-9995.2005.00774.x 15813816

[B44] Kakinuma T Nakamura K Wakugawa M Mitsui H Tada Y Saeki H Torii H Komine M Asahina A Tamaki K Serum macrophage-derived chemokine (MDC) levels are closely related with the disease activity of atopic dermatitis Clin Exp Immunol 2002 127 2 270 273 10.1046/j.1365-2249.2002.01727.x 11876749 PMC1906347

[B45] Uchida T Suto H Ra C Ogawa H Kobata T Okumura K Preferential expression of Th2-type chemokine and its receptor in atopic dermatitis Int Immunol 2002 14 12 1431 1438 10.1093/intimm/dxf109 12456591

