# Supplementary material for: Immunomodulatory effect of water soluble extract separated from mycelium of Phellinus linteus on experimental atopic dermatitis
Source: BMC Complement Altern Med. 2012 Sep 18;12:159. doi: 10.1186/1472-6882-12-159 (PMC3479073; doi:10.1186/1472-6882-12-159)

Supplement Figure 1. Water soluble extract of *P. linteus* (WA) inhibits the expression of pathogenic cytokines and chemokines under the antigen specific stimulation. AD-induced mice were sacrificed and ears were removed. Total ear cells were stimulated with WA (0.5 mg/ml) or ceramide (10 μg /ml) in the presence of mite extract (5 μg /ml) for 24 hrs, then mRNA expression of chemokines (A) and cytokines (B) were determined by quantitative RT-PCR. The mRNA expression levels of each sample were normalized with L32 (house- keeping gene) and then fold induction of each target gene were compared to PBS treated control. Error bars indicated S.D. One (*), Two (**) and three asterisk (***) indicates $P < 0.05$, $P < 0.005$, $P < 0.001$, respectively. Data are representative of three independent experiments.

Supplement Figure 1

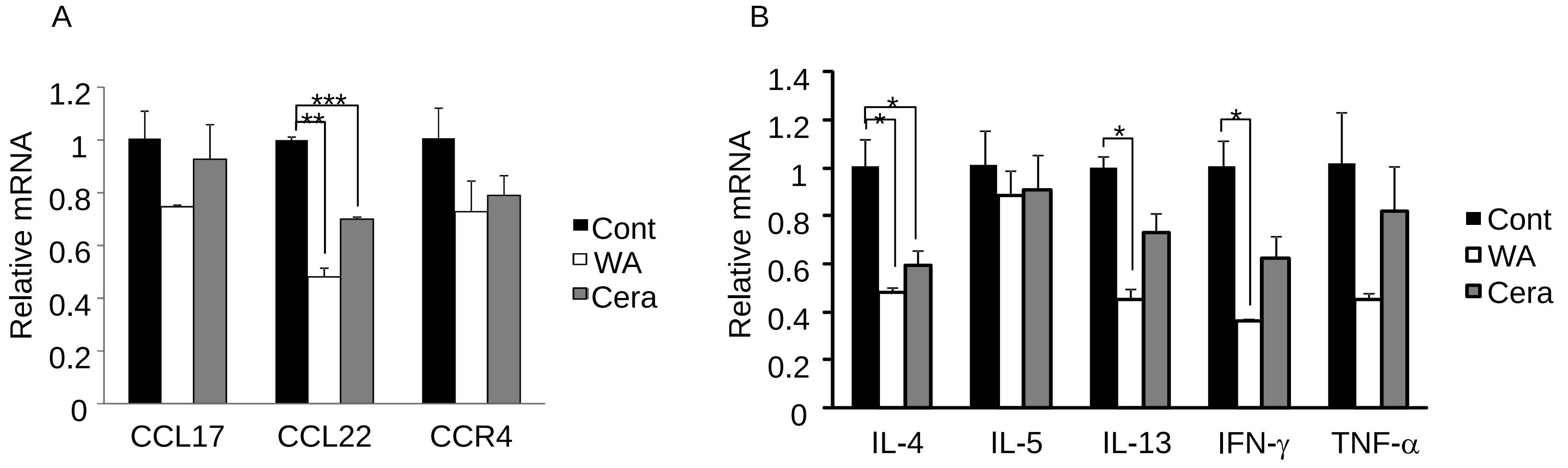

Supplement: Additional file 1 — Figure S1. Water soluble extract of P. linteus (WA) inhibits the expression of pathogenic cytokines and chemokines under the antigen specific stimulation. AD-induced mice were sacrificed and ears were removed. Total ear cells were stimulated with WA (0.5 mg/ml) or ceramide (10 μg /ml) in the presence of mite extract (5 μg /ml) for 24 hrs, then mRNA expression of chemokines (A) and cytokines (B) were determined by quantitative RT-PCR. The mRNA expression levels of each sample were normalized with L32 (house- keeping gene) and then fold induction of each target gene were compared to PBS treated control. Error bars indicated S.D. One (*), Two (**) and three asterisk (***) indicates P < 0.05, P < 0.005, P < 0.001, respectively. Data are representative of three independent experiments. [file 1472-6882-12-159-S1.pdf]
